# Better future with better us: Exploring young people's energy-saving behavior based on norm activation theory

**DOI:** 10.3389/fpubh.2022.1042325

**Published:** 2022-10-20

**Authors:** Hongyun Si, Ze Yu, Qi Jiang, Yimeng Shu, Wenwen Hua, Xiaoyan Lv

**Affiliations:** ^1^School of Public Administration and Policy, Shandong University of Finance and Economics, Jinan, China; ^2^School of Economics and Management, Tongji University, Shanghai, China; ^3^School of Management Science and Engineering, Shandong University of Finance and Economics, Jinan, China

**Keywords:** energy-saving behavior of young people, norm activation theory, PLS-SEM, self-efficacy, information publicity

## Abstract

Conserving energy use is a shared responsibility of all people, and it is essential for mitigating climate warming. The purpose of this study is to investigate energy-saving behaviors and the influencing factors of young people. We developed a new theoretical framework by adding self-efficacy and information publicity to norm activation theory. Partial least squares structural equation modeling was used to analyze 360 sample data from different regions in China. The findings show that attribution of responsibility and awareness of consequence are important prerequisites for personal norm. Personal norm positively influences energy-saving intention. Interestingly, information publicity has a significant positive effect on both intention and behavior to save energy, while self-efficacy only significantly affects energy-saving intention. This study focuses on the young group and enriches the research on factors influencing residents' energy-saving behaviors. The findings provide insightful ideas for governments and communities to guide individual energy conservation behaviors.

## Introduction

Since the 21st century, energy consumption has continued to grow all over the world (1). The global per capita energy consumption growth rate in 2018 is almost double that of 2010 (2), and *CO*_2_ emissions have increased by 40% globally from 2000 to 2019 (3). Energy conservation remains a serious global challenge, and it is difficult to solve the problems of high energy consumption only by promoting energy-saving technologies and clean energy (1). Conserving energy use is a shared responsibility of all people, and changing people's energy consumption habits becomes a feasible solution to energy and environmental problems (4, 5).

It is of great meaning to foster a sense of environmental concern for young people (6). In recent years, the growth in population numbers and urbanization levels has brought an increase in energy consumption. The demand for domestic energy by urban residents, especially the younger age groups, is expanding (7). As high energy consumers and potential major energy consumers in the future, young people are an important target group for energy-saving interventions (8). Young people's high receptiveness to information, their higher awareness of environmental protection and consequences, and their new personal norms make their behavior and willingness to save energy different from those of other age groups. Therefore, the energy-saving behavior (EB) of young people is the focus of this study with a certain research value.

Current research on the influencing factors of EB focuses on the fields of sociology and applied psychology. For example, Hong et al. (9) used a sociological approach to analyze the macro policy perspective and suggested that government subsidies can promote EB among residents. Wang et al. (10) combined sociological and psychological research to investigate EB in terms of monetary incentives for electricity prices and moral suasion. Although research on environmentally friendly behaviors is relatively mature, as a potential energy-saving subject, few studies have focused on related behaviors among young people. By organizing the findings, we can classify these factors into external intervention factors and internal individual factors. This study uses the extended Norm Activation Model (NAM) to empirically analyze young residents' energy-saving willingness and behavior based on external intervention factors and individual internal factors. The two extended variables of self-efficacy (SFE) and information publicity (IP) are integrated into NAM to reveal the influence path of EB on young residents (7).

## Literature review and hypothesis

### Related research about NAM

Currently, Theory of Planned Behavior (TPB), NAM, and Structural Equation Modeling (SEM) is used to conduct research related to the factors influencing altruistic behavior (11–13). NAM is a theoretical model for investigating altruistic behavior based on the personal norm (PN) (14). Pro-environmental behavior is often associated with PN, and problem perception and attribution of responsibility (AR) in NAM have very strong explanatory power for pro-environmental intentions (15). Thus, NAM is widely used to explain altruistic behavior and altruistic intentions. Related research topics include energy saving (16), water saving (17), and electricity saving behaviors (18).

Young people are a typical pro-environmental altruistic behavior that applies to NAM. We collated relevant studies applying NAM and its extended models in recent years (Table 1). Zhang et al. (16) used 297 validated interviews in Jinan, China, and explored the mechanisms of individual subjective, external influences and willingness on EB. Fu et al. (19) identified that cognitive attitudes influence people's willingness to save electricity based on TPB and NAM. Hien and Chi (20) showed that primary factors (e.g., perceived behavioral control, subjective norms, attitudes, and personal ethics) and additional factors (perceived benefits) in TPB and NAM are important factors that influence residents' willingness to save electricity. In addition, electricity saving behavior is influenced by willingness, perceived benefits, policy guidance and social advocacy. Du and Pan (21) indicated that PN has a significant positive effect on the willingness to save energy. Yang et al. (23) found that SFE and perceived control showed a significant positive effect on the intention to engage in habitual EB. The existing literature provides the foundation for exploratory research on EB, but at the same time, there has been insufficient attention to specific groups, such as younger age groups. Therefore, this study focuses on young people, considering the importance of SFE and IP, we extend these two variables to the NAM to reveal the determinants that influence young people's energy saving behavior.

**Table 1:** Recent literature on NAM and other extended factors.

**Literature**	**Context**	**Research subject**	**Theoretical basis**	**Expanded drivers**	**Significant results**
Zhang et al. (16)	Household energy consumption	EB of household	SEM	Energy efficiency policy	Individual factors, external factors and intentions act on EB.
Fu et al. (19)	Conservative energy	EB of household	NAM& TPB	Policy awareness	Residents' willingness to save electricity positively correlated with perceived behavioral control and personal ethics.
Hien and Chi (20)	Increase in global electricity demand	EB of residents	NAM& TPB	Policy and social publicize	The perceived behavioral control, subjective norm, personal moral, policies, and social propaganda benefits affect household electricity saving behavior.
Du and Pan (21)	Carbon emissions	EB of students	TPB	Personal ethics	Personal ethics are found to enhance interpretability of TPB.
Li et al. (22)	Carbon neutral	EB of residents	NAM& TPB	Environmental habituation	Environmental problems indirectly affect residents' willingness to save energy.
Yang et al. (23)	Carbon neutral	EB of college students	TPB	Environmental concern	Perceived SFE and perceived control mediators between information intervention and Energy-saving intention.
Zhang et al. (18)	Energy consumption	EB of employee power	NAM	Energy-efficient climate	PN have a positive impact on employees' EB.

### Research hypothesis

#### Extended NAM model

The original variable factors of NAM are Awareness of Consequence (AC), Attribution of Responsibility (AR), and Personal Norm (PN), of which PN is one of the core variables. PN affects behavioral intentions directly and has indirect effects through AC and AR. AC refers to whether individuals notice that pro-environmental behaviors can affect the person or object they are concerned about (24). In general, the stronger a person's perception of a particular outcome, then the stronger the moral obligation, and the more likely they are to activate their PN and display altruistic traits. AR refers to an individual's sense of responsibility for the results of performing pro-environmental behaviors (24). Cui et al. (25) found that awareness of the harmful outcomes of non-energy saving behaviors significantly influenced EB; AC and AR had a direct contribution to the formation of PN. Individuals realize that non-energy-saving behaviors can lead to serious environmental and social problems, thus promoting the formation of AR and PN, and thus adopting EB. For pro-environmental behaviors with altruistic characteristics, PN continuously generates positive intentions, which are an important prerequisite for the performance of altruistic behaviors (26). Thus, the willingness to save energy directly influences the EB. Therefore, the more users perceive the consequences, the greater their sense of responsibility and the more likely they are to activate self-regulation and thus be more willing to engage in energy-saving activities. Therefore, NAM is applicable to young people's interpretation and prediction of EB and willingness to save energy (EI). Based on the above, we propose the following hypotheses.

For this study, AC can be interpreted as young people's perceptions of the consequences of non-energy-efficient behavior on the adverse effects of environmental pollution, climate warming, and increased carbon emissions. NAM notes that when young people believe that their non-energy-efficient behavior is causing negative environmental impacts, they are more likely to attribute these consequences to themselves and thus fulfill their moral obligations and responsibilities. Therefore, NAM is applicable to young people's interpretation and prediction of EB and energy-saving intention (EI). Based on the above, we propose the following hypotheses:

H1. Among young people, AC has a significant effect on PN.
H2. Among young people, AR has a significant effect on PN.
H3. Among young people, PN has a significant effect on EI.
H4. Among young people, EI has a significant effect on EB.

#### Self-efficacy

In NAM, SFE is used as the core variable to influence the willingness as well as the behavior to save energy. SFE is the confidence to accomplish a certain behavior, and the level of SFE affects an individual's behavioral decisions (27). Yang et al. (23) demonstrated that SFE and perceptual control had a significant positive effect on EI in the group of college students. Wang et al. (28) found that SFE directly influenced energy-saving-related behaviors in a study of office workers in the Netherlands. Allen and Marquart-Pyatt (29) reached similar conclusions in their study of the energy conservation behaviors of campus community members. In the case of residential EB, young individuals are directly influenced by SFE when making decisions about EB. SFE also influences EB through EI. It leads to the following hypothesis for this study:

H5. Among young people, SFE has a significant effect on EI.
H6. Among young people, SFE has a significant effect on EB.

#### Information publicity

Information publicity (IP) is usually defined as the process of transmitting information to individual units through specific policies, documents and other means of communication by governments, businesses, communities and specific organizations (30). In this study, we include IP as an extended variable in NAM. It is the process of government, organizations and communities promoting EB through online advertising and offline display boards. Yue et al. (30) found that policies and measures that promote the culture of energy conservation can help the public save energy more effectively. The correlation between EB and their influences suggests that motivation at the spiritual and material levels is equally important. Xu et al. (31) found from an analysis of questionnaire surveys conducted in four megacities (Beijing, Hangzhou, Guangzhou, and Guiyang) that energy conservation education should be enhanced to raise residents' awareness and thus promote EB. Wang et al. (32) found that IP has a strong influence on EB when they studied the daily behavior of Chinese urban residents. Among young groups, the intervention of external information influences individual decisions (32, 33). In specific scenarios, young people's decisions about EB are influenced by external information campaigns, which in turn influence their willingness and behavior to save energy. From this, we propose the following hypothesis.

H7. Among young people, IP has a significant effect on EB.
H8. Among young people, IP has a significant effect on EI.

**Figure 1:**
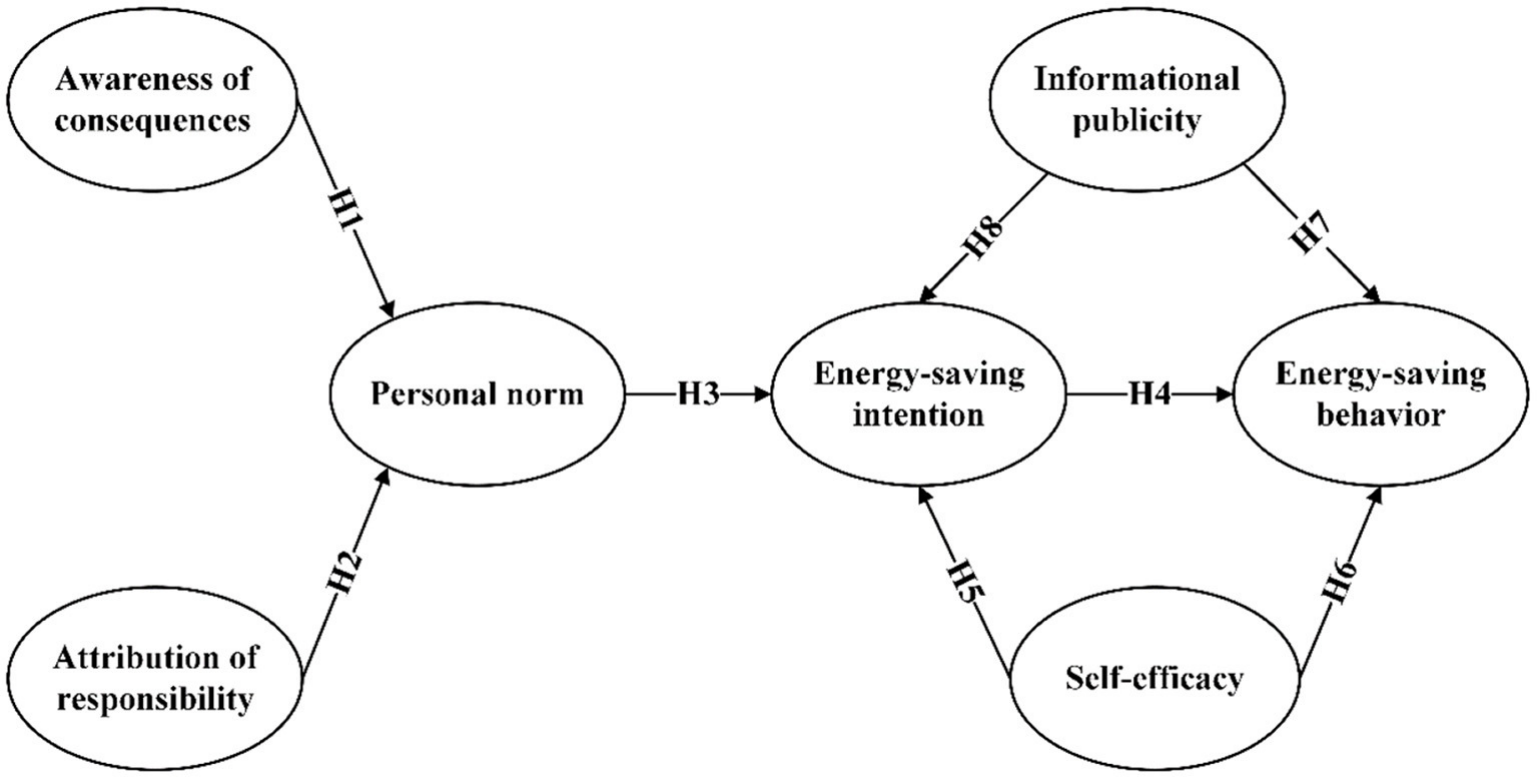
Energy-saving behavior model.

The conceptual model of this study is shown in Figure 1.

## Methodology

### Partial least squares structural equation model

This study uses partial least squares structural equation modeling (PLS-SEM) for empirical analysis to explore the factors that influence the willingness and behavior to save energy among the younger age group of residents. An extended NAM is used to hypothesize the possible paths that influence the willingness and behavior to save energy. Unlike conventional SEM, which only constructs reflective structural models, PLS-SEM can construct both reflective and formative measurement models (13). PLS-SEM is a multivariate data processing tool that can verify or predict the relationship between variables without the data following a normal distribution (34). Thus, it applies to several fields, such as public transportation management (35), customer satisfaction and loyalty in sociology (36), vaccination intentions in psychology (37), and predictive model evaluation in engineering (38). In this study, a comprehensive analysis was conducted for internal and external factors of EB, and then the relationship between the predictor variables was verified to analyze the influencing factors that affect young people's EB and EI. In summary, PLS-SEM is feasible for this study.

### Survey design

The questionnaire for this study consisted of three parts. The first part was a description, which included the survey purpose and the explanation of EB in this study. The second part investigated the socio-demographic characteristics of the participants, including gender, age, education level, income, and occupation. The third part included question items measuring eight latent variables.

The measurement items in this questionnaire mainly referred to previous studies and were modified according to the content of this study. The questionnaire design was pretested and insignificant measurement items were removed, and 29 valid measurement items were finally identified. The questionnaire was designed using a 7-point Likert scale, with one indicating complete disagreement and seven indicating complete agreement. The 7-point scale provided more options for the research respondents compared to the 5-point Likert scale, which improved the reliability of the data, and Table 2 shows the specific measurement questions for the latent variables in detail.

**Table 2:** Description of construct and measurement items.

**Construct**	**Measurement item**	**Mean** **(standard deviation)**	**References**
AC	The energy non-conservation behavior of residents may lead to damage to the ecosystem.	5.7005 (1.5890)	(18, 39)
	Inefficient energy use by residents may lead to global warming.		
	Residents' failure to save energy may lead to severe environmental pollution.		
	Residents' energy-saving behavior can reduce carbon emissions.		
AR	I am responsible for global warming caused by non-energy-saving behavior.	5.0612 (1.7368)	(18, 20)
	I am responsible for the damage to the ecological environment caused by the non-energy-saving behavior.		
	I am responsible for the severe environmental pollution caused by the non-energy saving behavior.		
	I am responsible for the increase in carbon emissions caused by non-energy saving behavior.		
SFE	I feel that my energy-saving behavior can set an example for others.	5.6047 (1.3893)	(23, 40)
	I feel that my energy-saving behavior can promote the sustainable development of the city to a certain extent.		
	I feel that my energy-saving behavior can help achieve carbon reduction.		
PN	When the residents' non-energy-saving behavior occurred, I felt guilty.	5.9193 (1.2807)	(19, 21)
	I think it is essential to save energy as a resident.		
	I will take the initiative to buy energy-saving products.		
EB	I will actively turn off the air conditioner or adjust to energy-saving mode.	5.7363 (1.4045)	(41, 42)
	I will take the initiative to avoid setting the electric water heater to low-power insulation for a long time.		
	I would turn off the electrical equipment before leaving the empty room.		
	I am willing to save energy in my daily life.		
EI	I intend to engage in energy-saving activities in my daily life.	5.9418 (1.2276)	(16, 43)
	I am willing to participate in some energy conservation and emission reduction activities.		
	I am willing to buy energy-saving products.		
	I am willing to respond to the energy-saving policy.		
	I think it is essential to disseminate information related to energy conservation.		
IP	Publishing information about the current state of energy will encourage me to conserve energy.	5.8440 (1.2903)	(30, 31)
	Spreading the word about energy conservation will motivate me to save energy.		
	I can see energy-saving advertisements through online channels such as the Internet, motivating me to save energy.		
	In reality, I will be inspired to save energy because of the energy-saving advertising display board.		
	The government and environmental protection departments will influence my electricity consumption behavior.		

### Data collection

**Figure 2:**
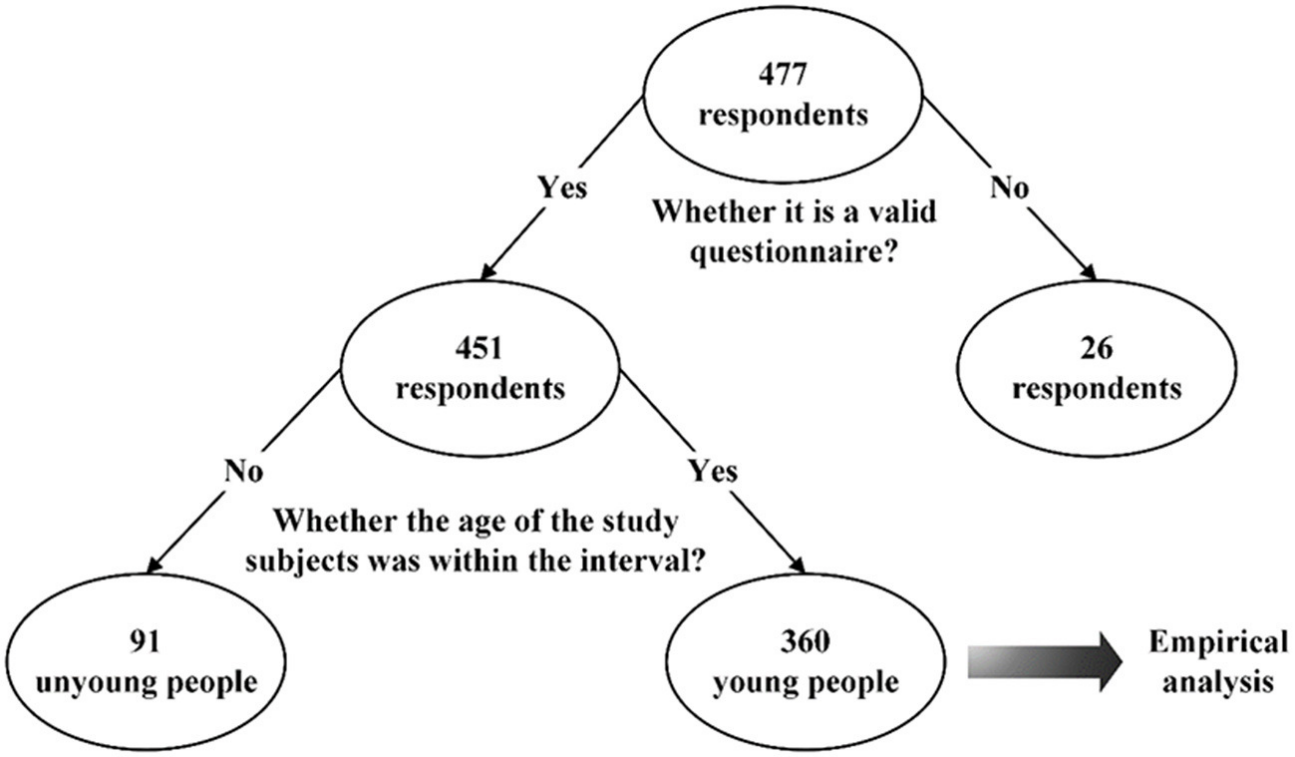
Data screening process.

The data for this study were collected through the Chinese questionnaire platform, Wenjuanxing. Wenjuanxing (44) is a professional online questionnaire platform that is widely used in the data collection phase of empirical studies. The questionnaire data were collected using a national scale sample database, and target populations from different regions of China were invited to complete the questionnaire. The division of the young group was referred to the United Nations World Health Organization, and people under 35 years old were taken as the target population of the survey (8). We collected a total of 477 online questionnaires. Twenty six invalid questionnaires were excluded based on trap items and preliminary screening, and 451 valid questionnaires were obtained, of which the sample size of the young group that fit the study population was 360. The detailed data screening process is shown in Figure 2, and the specific sociodemographic characteristics are shown in Table 3.

**Table 3:** Socio-demographic information of respondents.

**Characteristic**	**Demographic information**	**Frequency**	**%**
Gender	Male	151	41.94
	Female	209	58.06
Age	Under 20	150	41.67
	21–25	80	22.22
	26–30	43	11.94
	31–35	86	23.89
Educational	High school or lower	43	11.94
level	Junior college	40	11.11
	Undergraduate university degree	251	69.72
	Postgraduate university degree	26	7.220
Monthly	< 2,000	179	49.72
income (CNY)	2,000–4,000	55	15.28
	4,000–6,000	40	11.11
	6,000–8,000	39	10.83
	Over 8,000	47	13.06
Occupation	The staff of administrative bodies	13	3.610
	Public institution staff	26	7.220
	Employees of private enterprises	68	18.89
	Freelancer	38	10.56
	Farmer	1	0.280
	College students	214	59.44

According to Table 3, among the 360 valid participants, most of those who completed the questionnaire were female (58.06%) and 41.94% were male. The age of the study sample after data screening was <35 years, which meets the criteria for young people set by the United Nations. In addition, the majority of the study respondents had education levels of college and above (88.06%), had a monthly income of <2,000 (CNY) (49.72%), and had students as their primary occupation (59.44%), which is in line with the research objectives for the pro-environmental behavior of the younger group. The young college student population is influenced by multiple factors in their EB (45), and the causes are more complex (7). In summary, the sample fits the target group characteristics of our study.

## Results analysis

### Evaluation of the measurement model

We used Cronbach's Alpha (CA), Composite Reliability (CR) and Average Variance Extracted (AVE) to test the reliability of the survey data. The results of the reliability evaluation are shown in Table 4. The CA values of the survey data models in this study were all above 0.82, indicating good reliability (46). The CR values for each variable ranged from 0.898 to 0.978, which was greater than the critical value of 0.7 (47), indicating good internal consistency of the scales used for each variable. The AVE values of the data ranged from 0.733 to 0.918, which exceeded the criterion of 0.5 (46), indicating that the measurement model had sufficient convergent validity. The results of CA values and CR values indicate that the model has good internal consistency and high reliability, and the questionnaire data meet the needs of the study.

**Table 4:** Reliability and convergence validity testing results.

**Item**	**Cronbach's alpha**	**Composite reliability**	**Average variance extracted**
AC	0.899	0.931	0.773
AR	0.970	0.978	0.918
EB	0.878	0.916	0.733
IP	0.933	0.950	0.790
PN	0.945	0.956	0.783
SFE	0.829	0.898	0.746
EI	0.905	0.941	0.841

Table 5 shows the test results of discriminant validity based on the measurement model of the Fornell and Larcker (48), where the data values in the first vertical row (bold) indicate the square root of AVE and the remaining data values indicate the correlation coefficients of the different constructs. The model has good discriminant validity (48) when all square roots of AVE (all bolded data values) are greater than all other data values in the row and column where the value is located. Table 6 shows the factor loadings of the measured question items and the cross-loadings of the other latent variables. The bolded data values represent the latent variables. The factor distribution of all latent variables ranged from 0.760 to 0.964, which was greater than the standard value of 0.6 and exceeded the cross-loadings of the other constructs (49), and the discriminant validity of the model can be judged to meet the requirements.

**Table 5:** Discriminant validity testing results.

	**AC**	**AR**	**EB**	**EI**	**IP**	**PN**	**SFE**
AC	**0.879**						
AR	0.456	**0.958**					
EB	0.407	0.405	**0.856**				
EI	0.472	0.432	0.787	**0.889**			
IP	0.495	0.474	0.754	0.868	**0.885**		
PN	0.565	0.610	0.609	0.704	0.723	**0.864**	
SFE	0.479	0.551	0.634	0.714	0.705	0.644	**0.917**

The bold values indicate the square root of AVE.

**Table 6:** Factor loading (bold font) and cross-loading.

	**AC**	**AR**	**EB**	**EI**	**IP**	**PN**	**SFE**
AC1	**0.915**	0.430	0.381	0.442	0.450	0.521	0.417
AC2	**0.913**	0.405	0.360	0.378	0.400	0.470	0.412
AC3	**0.918**	0.419	0.393	0.443	0.472	0.498	0.434
AC4	**0.760**	0.343	0.290	0.389	0.413	0.490	0.416
AR1	0.464	**0.958**	0.413	0.434	0.468	0.600	0.550
AR2	0.430	**0.964**	0.402	0.429	0.465	0.572	0.520
AR3	0.412	**0.955**	0.380	0.414	0.453	0.576	0.522
AR4	0.440	**0.957**	0.357	0.380	0.431	0.589	0.517
EB1	0.407	0.395	**0.836**	0.754	0.680	0.580	0.566
EB2	0.345	0.391	**0.893**	0.698	0.650	0.507	0.611
EB3	0.305	0.349	**0.891**	0.651	0.639	0.499	0.537
EB4	0.327	0.232	**0.800**	0.572	0.608	0.492	0.439
EI1	0.439	0.342	0.763	**0.864**	0.751	0.671	0.604
EI2	0.386	0.394	0.712	**0.917**	0.789	0.621	0.711
EI3	0.382	0.395	0.650	**0.882**	0.776	0.571	0.662
EI4	0.416	0.411	0.716	**0.901**	0.769	0.625	0.628
EI5	0.476	0.380	0.650	**0.880**	0.773	0.640	0.564
IP1	0.436	0.384	0.709	0.810	**0.879**	0.676	0.613
IP2	0.503	0.411	0.699	0.820	**0.904**	0.671	0.617
IP3	0.450	0.400	0.681	0.800	**0.911**	0.668	0.630
IP4	0.371	0.441	0.614	0.689	**0.863**	0.593	0.628
IP5	0.403	0.427	0.625	0.695	**0.870**	0.593	0.616
IP6	0.457	0.460	0.667	0.780	**0.883**	0.630	0.643
PN1	0.442	0.536	0.472	0.549	0.518	**0.809**	0.522
PN2	0.499	0.540	0.575	0.650	0.683	**0.883**	0.567
PN3	0.519	0.506	0.527	0.621	0.665	**0.897**	0.577
SFE1	0.408	0.482	0.584	0.652	0.646	0.587	**0.896**
SFE2	0.448	0.504	0.586	0.653	0.647	0.564	**0.937**
SFE3	0.461	0.529	0.573	0.659	0.648	0.620	**0.918**

The bold values indicate the factors of the latent variables.

In this study, the variance inflation factor (VIF) of the internal and external models was calculated by PLS. When the VIF is <10, it indicates that there is no covariance problem between the independent variables (34). The VIF values of all the observed factors in this study range from 1.262 to 8.377, all of which are <10, implying that the internal and external structures do not have multicollinearity.

### Evaluation of the structural model

**Figure 3:**
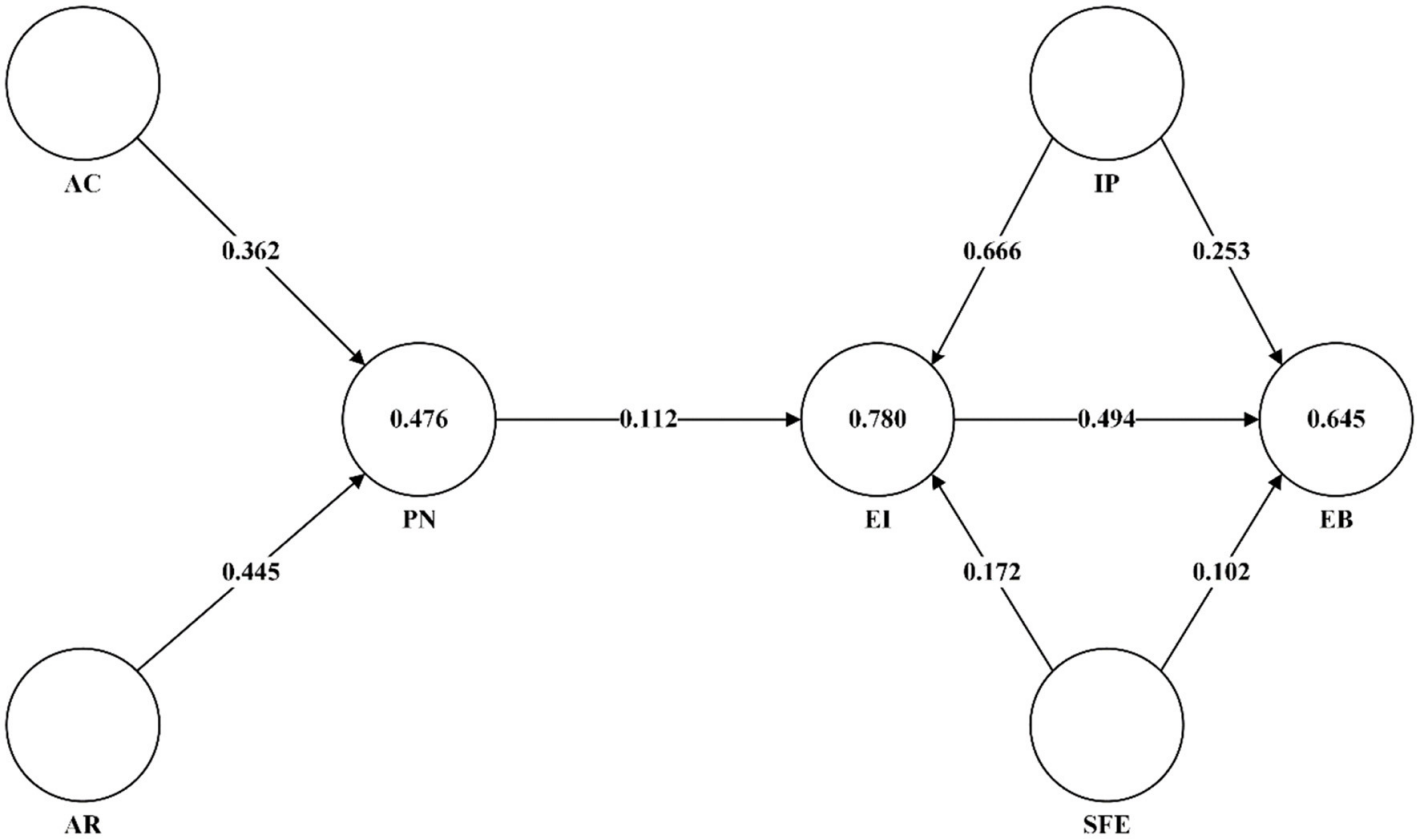
Model path coefficients, *R*^2^ (inside the circle) values.

*R*^2^ measures the fit degree of the predicted value to the true value (46). The *R*^2^ of PN, EI, and EB in this study are the values in the circles in Figure 3. The *R*^2^ of PN is 0.476, indicating that 47.6% of the variance in PN is explained jointly by AC and AR. the *R*^2^ of EI is 0.781, indicating that 78.1% of the variance in EI is explained jointly by SFE, PN, and IP. The *R*^2^ value for EB is 0.645, indicating that 64.5% of the variance in EB is explained by SFE, IP and EI together. In consumer-related studies, the value of *R*^2^ above 0.20 is considered a higher adjudicated value that adequately explains the variance in the model (13). In summary, the theoretical model of this study has good explanatory power for PN, EI and EB of the younger group of residents.

**Figure 4:**
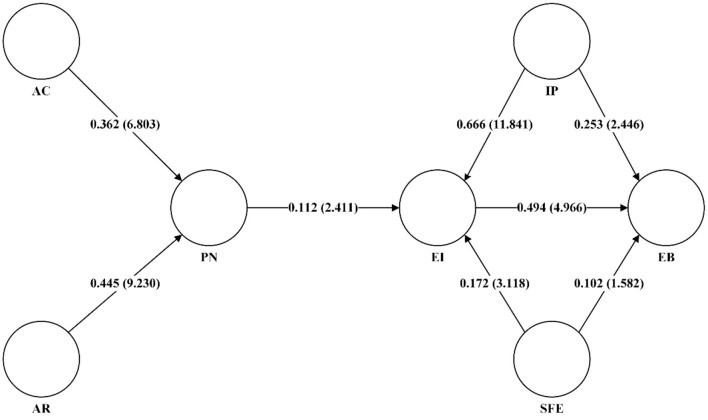
Model path coefficients and T values (in parentheses).

The eight main effects tests were conducted to test the direct paths between AC, AR, IP, SFE, PN, EI, and EB in the model, as shown in Figure 4 and Table 7. The model results showed that all the hypotheses were verified except H6. And the results of the confidence intervals of the eight paths in this study are consistent with the above findings, and it can be concluded that the model shows good statistical significance.

**Table 7:** Empirical results of hypotheses.

	**Path**	**Standardized**	**T Statistics**	***P*-values**	**Confidence interval**		**Hypothesis**
		**Path coefficient**			**2.50%**	**97.50%**	**Test**
H1	AC -> PN	0.362	6.803	0.000	0.257	0.470	Supported
H2	AR -> PN	0.445	9.230	0.000	0.352	0.535	Supported
H3	PN -> EI	0.112	2.411	0.016	0.02	0.203	Supported
H4	EI -> EB	0.494	4.966	0.000	0.306	0.707	Supported
H5	SFE -> EI	0.172	3.118	0.002	0.057	0.268	Supported
H6	SFE -> EB	0.102	1.582	0.114	−0.024	0.231	Not supported
H7	IP -> EB	0.253	2.446	0.015	0.039	0.426	Supported
H8	IP -> EI	0.666	11.841	0.000	0.546	0.773	Supported

## Discussion

###  Results discussion

The survey results reflect the current status of EB among young people in China. According to the results of PLS-SEM, the original hypothesis of NAM is supported and consistent with the results of previous studies on EB of urban residents. First, AC positively affects PN, a finding identical to that obtained in Zhang et al. (18) study of employees' EB. AR has a significant positive effect on PN, which is consistent with the original hypothesis of NAM (24). As Hong et al. (9) mentioned in their study, urban residents' sense of environmental responsibility has a significant positive effect on PN, i.e., AC has a significant effect on PN, while Han and Cudjoe (41) argued that AC indirectly affects PN. In our study, this finding that AC affects PN was again validated in the younger age group. In addition, many studies have concluded that PN has a significant positive effect on EI and EB. For example, Li et al. (22) found that PN affects the willingness of residents to engage in habitual EB in a study on environmental issues. This result remains consistent with the results of this study. That is, in EB of the young group, the sense of responsibility for the environment and the awareness of the consequences of non-energy-saving behavior activate the individual's PN, which in turn promotes EB. In summary, the hypotheses of the original model of NAM were all validated in this study, reasonably explaining the factors influencing EB of young people, and validating the reasonableness of the prior variables of the original model of NAM.

In addition, another variable that was extended in this study was SFE. The findings of this study showed a significant effect of SFE on EI. The present results are consistent with the results of previous studies. For example, Yang et al. (23) suggested that perceived SFE and perceived control have a significant positive effect on habitual energy-saving behavioral intentions in urban residents, i.e., the effect of SFE on EI. But surprisingly, the hypothesis that SFE has a significant effect on EB in this study did not hold, which was not consistent with previous studies, where Yang et al. (23) concluded that SFE affected EB in urban residents. By analyzing the behavioral characteristics of young people, we learned that young people have a weaker sense of SFE compared to other age groups and that their EB is more influenced by, for example, IP or financial benefits. So it may cause that the direct effect of SFB on EB here is not significant. Fatmawati et al. (7) also concluded in a study of EB among young people in Indonesia that the participants showed good attitudes, willingness, and behavioral SFE toward electricity-saving behavior. However, the findings of this study are consistent with the findings of Foster et al. (40) on the pro-environmental behavior of 150 public officials in an organization in Terengganu. The possible reason for the inconsistent results is that the study respondents did not pay enough attention to EB itself, while the reason for the consistency with the results of Foster et al. (40) study may be that more of their respondents were young people. According to Fatmawati et al. (7), the younger group belonged to those with the lowest energy consumption rate and low attention to EB. Moreover, this group was influenced by life experiences and maybe positively motivated to save energy when they had a sufficiently high SFE, but not enough to support the implementation of EB. The above results suggest that SFE, as an individual's confidence to accomplish a certain behavior, can directly and positively influence EI, but does not directly influence EB. In summary, the extended variables in this study can also have a significant positive effect on EB, further enriching the variables of NAM. However, we also found that SFE did not directly affect EB significantly. The results of the study provide some references for the development of related policies. And IP had a positive impact on EB in this study. This finding is consistent with previous studies, e.g., Fatmawati et al. (7) found that IP was of research value in a study of EB among young people, and Wang et al. (33) found that community campaigns can improve electricity savings in a study of community EB. These findings fit with the behavioral characteristics of young people. Young people receive a wider range of information and are more receptive. In addition, the measurement questions in this study also demonstrate that increased information dissemination on EB by government, community, and social organizations, both online and offline, can help promote EB among residents. In summary, IP can positively influence young people's EI and EB. The findings of this study further enrich the factors that influence young people's EB.

The contributions of this study are as follows. First, this study extends the NAM's explanation of adolescents' EB. The subjects of this study are young people, and as adolescents are important potential targets for implementing EB, conducting a study on youth is strategically important for implementing carbon reduction, and it is conducive to further promoting the implementation of EB among residents on a larger scale. Second, this study further strengthens the explanatory ability of the NAM and expands its use boundaries. The validity of the expanded model was verified by integrating two extended variables, SFE and IP, into the NAM, confirming the application of the NAM in the field of altruistic behavior. In addition, the results provide feasible policy suggestions for governments, communities, and relevant organizations, and serve as a reference for the research of EB in other countries and regions.

### Policy implications

This study used the young group of residents as the study population and concluded that IP, PN, and SFE played a significant and positive role in influencing the young group's willingness and behavior to save energy. Corresponding policy implications include that descriptive norms and prohibitions of EB should be strengthened first. On the one hand, government, community and other social organizations can conduct education related to energy conservation to promote awareness of energy efficiency policies among younger groups. The government can use exemplary energy-saving communities as examples and call on other communities to follow suit. On the other hand, the government can further motivate SFE of the research subjects by encouraging and recognizing EB in young people, so that the young people will have a stronger desire to save energy and thus promote EB.

More awareness campaigns should be developed to promote the willingness and behavior of young residents to save energy. Given that young people are more receptive to information on the Internet, they can use online resources and online channels to promote EB. Self-media channels such as short videos can be used to promote EB and related policies. Collaborate with public figures or Vloggers to call on city residents to join in energy-saving initiatives. At the same time, we should also focus on offline publicity and the function of public display boards to expand the coverage of EB. At the same time, we can also organize energy-saving activities on university campuses, and have the government and schools organize public welfare activities, so that young people such as college students can actively participate in EB and maximize the positive results of EB for the whole society and the environment.

## Conclusions and limitations

### Conclusions

The purpose of this study was to examine the factors influencing EB among the younger age group of the residents. Using data from 360 questionnaires obtained in China, an extended NAM was obtained by expanding SFE and IP into NAM. The results of this study also verify the applicability of this extended model. Based on PLS-SEM, it is verified that the extended NAM has strong explanatory power for EI and EB of the study subjects. The empirical results show that AR and AC in young people's EB are important prerequisites for PN. Both PN and IP have significant positive effects on the study subjects' EI and EB. SFE has a significant direct effect on EI, but SFE does not have a significant direct effect on EB. Overall, external intervention factors in the younger group have a greater impact on EI than internal psychological factors (these factors were IP, SFE, and PN, in descending order of importance). That was, external intervention factors play an important role in EB of the young group. Based on these findings, we provide policy recommendations for the government, communities, and related organizations to further promote EB among young people.

### Limitations and future research directions

Although we have new findings, several limitations of this study should still be noted. First, although we used an extended NAM, the influencing factors that affect people's EB in life are more complex. Subsequent studies can continuously extend the model to add more influencing factors. For example, subjective norms, attitudes, perceived behavioral control, social norms, and energy-saving climate. Second, this study used a questionnaire survey. Future studies should expand the sample size and further improve the representativeness of the sample. Also, this study should refine the socio-demographic characteristics and conduct a more detailed multi-group analysis. For instance, comparisons between different age groups, different genders, and different education levels of the study subjects could be added. Finally, future studies may be conducted in other parts of the world, taking into account differences in global energy environments, cultures, and economic levels.

## Data availability statement

The original contributions presented in the study are included in the article/supplementary material, further inquiries can be directed to the corresponding author/s.

## Author contributions

HS: conceptualization, methodology, and supervision. ZY: writing—original draft, data curation, validation, and formal analysis. QJ: methodology, editing, and supervision. YS: data curation, validation, and investigation. WH: writing—reviewing and editing, language enhancement, and supervision. XL: conceptualization, data curation, and writing—reviewing and editing. All authors contributed to the article and approved the submitted version.

## Funding

This work was supported by the National Natural Science Foundation of China (Grant Number: 72104128), the Key R&D Plan of Shandong Province of China (Soft Science Project, Grant Number: 2021RKY07133), the Humanities and Social Science Foundation of the Ministry of Education of China (Grant Number: 21YJC630117), the Natural Science Foundation of Shandong Province, China (Grant Numbers: ZR2021QG053 and ZR2021MG004), and the Youth Entrepreneurship Talent Introduction and Education Team of Colleges and Universities in Shandong Province, China.

## Conflict of interest

The authors declare that the research was conducted in the absence of any commercial or financial relationships that could be construed as a potential conflict of interest.

## Publisher's note

All claims expressed in this article are solely those of the authors and do not necessarily represent those of their affiliated organizations, or those of the publisher, the editors and the reviewers. Any product that may be evaluated in this article, or claim that may be made by its manufacturer, is not guaranteed or endorsed by the publisher.

## Abbreviations

AC: awareness of consequences
AR: attributing responsibility
AVE: average variance extracted
CA: Cronbach's alpha
CR: composite reliability
EB: energy-saving behavior
EI: energy-saving intention
IP: informational publicity
NAM: norm activation model
PLS: partial least squares
PN: personal norm
SEM: structural equation modeling
SFE: self-efficacy
VIF: variance inflation factor.
